# Functional analysis of the ATP-binding cassette (ABC) transporter gene family of *Tribolium castaneum*

**DOI:** 10.1186/1471-2164-14-6

**Published:** 2013-01-16

**Authors:** Gunnar Broehan, Tobias Kroeger, Marcé Lorenzen, Hans Merzendorfer

**Affiliations:** 1 Department of Biology/Chemistry, Animal Physiology, University of Osnabrück, Osnabrück, 49069, Germany; 2 Department of Entomology, North Carolina State University, Raleigh, NC, 27695, USA

**Keywords:** ABC transporter, Coleoptera, dsRNA, Genome, RNA interference, Red flour beetle, *Tribolium castaneum*

## Abstract

**Background:**

The ATP-binding cassette (ABC) transporters belong to a large superfamily of proteins that have important physiological functions in all living organisms. Most are integral membrane proteins that transport a broad spectrum of substrates across lipid membranes. In insects, ABC transporters are of special interest because of their role in insecticide resistance.

**Results:**

We have identified 73 ABC transporter genes in the genome of *T. castaneum*, which group into eight subfamilies (ABCA-H). This coleopteran ABC family is significantly larger than those reported for insects in other taxonomic groups. Phylogenetic analysis revealed that this increase is due to gene expansion within a single clade of subfamily ABCC. We performed an RNA interference (RNAi) screen to study the function of ABC transporters during development. In ten cases, injection of double-stranded RNA (dsRNA) into larvae caused developmental phenotypes, which included growth arrest and localized melanization, eye pigmentation defects, abnormal cuticle formation, egg-laying and egg-hatching defects, and mortality due to abortive molting and desiccation. Some of the ABC transporters we studied in closer detail to examine their role in lipid, ecdysteroid and eye pigment transport.

**Conclusions:**

The results from our study provide new insights into the physiological function of ABC transporters in *T. castaneum,* and may help to establish new target sites for insect control.

## Background

ATP-binding cassette (ABC) transporters constitute one of the largest protein superfamilies of integral membrane proteins. They are ubiquitously found in all kingdoms of life, where they typically function in the ATP-dependent transport of various substrates across biological membranes. The spectrum of transport substrates covers inorganic ions, sugars, amino acids, lipids, lipopolysaccharides, peptides, metals, xenobiotics and chemotherapeutic drugs [1]. In addition, ABC proteins can act as ion channels, ion channel regulators and receptors, and some have special roles in ribosomal assembly and translation. ABC transporters have a modular structure, which consists of four functional units: two nucleotide-binding domains (NBDs), which bind and hydrolyze ATP, and two transmembrane domains (TMDs), which are involved in translocation of the respective substrate [2]. ABC transporters that function as exporters are found in eukaryotes and prokaryotes, whereas ABC importers seem to be restricted to prokaryotes. The prokaryotic ABC importers exhibit an additional functional unit, a substrate-binding protein (SBP). An SBP has an high affinity for a specific substrate and delivers it to the ABC transporter where it is released into the translocation pore after ATP is bound and hydrolyzed by the NBDs [3].

NBDs of all ABC transporters contain three highly conserved motifs: Walker A, Walker B and an ABC “signature sequence” (C motif) located in between [4]. There are two schemes by which the domains of eukaryotic ABC transporters are organized. Either they are full-transporters combining all four domains (2 TMDs and 2 NBDs) in a single polypeptide, or they are half-transporters, consisting of only two domains (1 TMD and 1 NBD), which can be arranged either as TMD-NBD or NBD-TMD. The half-transporters need to form homo- or heterodimers to form a functional pump. Sequence analyses of eukaryotic ABC transporters revealed that they can be divided into eight subfamilies (ABCA-H). Subfamily ABCH, which is most closely related to subfamily ABCG, was identified first in the *Drosophila melanogaster* genome. ABCH genes appear to be present in all insects, *Dictyostelium* and zebrafish, but is absent from plants, worms, yeast, or mammalian genomes [5]. The first eukaryotic ABC transporter identified was the P-glycoprotein on the cell surface of cancer cells [6], which acts as a multidrug resistance (MDR) efflux transporter that prevents the accumulation of chemotherapeutic drugs. Since then, numerous ABC transporters have been shown to be involved in transport of various substrates in a wide range of eukaryotic organisms. Examples include Ste6 from the baker’s yeast *Saccharomyces cerevisiae*, which is involved in the export of the a-factor necessary for mating [7], Cer5 from *Arabidopsis thaliana*, which functions in the export of cuticular lipids [8], half-molecule ABC transporters of the HMT-1 subfamily from *Caenorhabditis elegans*, which convey tolerance to heavy metal ions [9], or the transporter associated with antigen processing (TAP) from humans, which delivers peptides to the major histocompatibility complex I (MHC I) for antigen presentation [10].

The physiological functions of ABC transporters in insects are presumably as manifold as implied for the different organisms above. They are involved in the transport of eye pigments [11], lipid-modified peptide chemoattractants [12], glutathione-conjugated organic anions [13], and possibly 20-hydroxyecdysone to orchestrate circadian transcription of clock genes [14]. More important from an economical point of view is the fact that many insect ABC transporters mediate tolerance to xenobiotics. The tobacco hornworm (*Manduca sexta*), for instance, can feed on tobacco leaves without getting poisoned by the neurotoxin nicotine, which is efficiently excreted by P-glycoprotein-like multidrug transporters in the Malpighian tubules [15]. From studies in different systems it becomes increasingly evident, that ABC transporters may contribute to insecticide resistance by countering the intracellular accumulation of insecticides and their metabolites [16]. Moreover, an ABC transporter was recently identified that may be crucial for the mode of action of Bt toxins. Mutations in the transporter confer high levels of resistance to Bt, which could pose serious problems for Bt-crops [17]. In addition, the sulfonylurea receptor (SUR), a subfamily C protein, has been suggested to be the target of benzoylurea-derived insecticides such as diflubenzuron [18]. Insect genome sequencing projects allowed the establishment of complete inventories of ABC transporters for *D. melanogaster* and the silkworm *Bombyx mori*[19-21]. However, a genome-wide functional analysis of genes encoding ABC transporters has not yet been performed for a coleopteran insect species. The red flour beetle, *Tribolium castaneum*, is an important stored product pest and a well-established genomic insect model allowing analysis of gene function by systemic RNAi [22].

To characterize the ABC transporter gene family of *T. castaneum*, we analyzed the entire sequenced genome to identify potential members of each subfamily. We identified 73 *Tribolium* genes encoding putative ABC transporters with members in all established ABCA-H subfamilies. Sequence comparisons of each subfamily member with available sequences from other insects revealed phylogenetic relationships. Next, we used RNA interference to individually knock down transcript levels of each of the 73 genes in penultimate larvae, and monitored further development to screen for evident phenotypes that could link the respective gene with a specific function. Using this approach we identified ten genes that lead to detectable phenotypes during development. Two of these are associated with the transport of eye pigments, two are related to the control of protein biosynthesis, two appear to have functions in lipid transport affecting waterproofing of the epicuticle, one seems to be involved in an ecdysteroid related processes, and another may have a developmental function. We demonstrate that an integrated analysis of ABC genes can provide insights on the physiological function of ABC transporters.

## Methods

### Insects

All analyses were carried out with the *Tribolium castaneum* strain GA-1 [23]. The beetles were reared on whole wheat flour containing 5% dried brewers’ yeast under standard conditions at 30°C as described previously [24].

### Bioinformatic analysis of the *Tribolium* ABC transporter superfamily

To identify open reading frames (ORFs) encoding putative ABC transporters, we conducted tblastn searches on the *Tribolium castaneum* 3.0 genome assembly (Tcas 3.0) using the orthologous groups protein database at BeetleBase (http://BeetleBase.org) [25]. As a query, we used highly conserved NBDs from the *Drosophila*, *Anopheles, Bombyx* and *Daphnia* genomes, whose ABC transporter superfamilies were characterized previously [19-21,26,27]. Hits from individual subfamilies were only considered when the E-values were less than 10^-6^. The sequences were re-evaluated with NCBI’s CDS and CDART programs (http://www.ncbi.nlm.nih.gov), as well as the probabilistic profile hidden Markov models (HMMs) using the HMMER webserver (http://hmmer.janelia.org). To assign putative *Tribolium* ABC genes to ABC subfamilies, the NBDs of the corresponding GLEAN models were extracted and used in ClustalW alignments. Next, phylogenetic trees were reconstructed with the maximum-likelihood method (5000 replicates for bootstrapping) using the MEGA 5.03 software [28]. Subfamily-specific clustering was then compared with that of previous phylogenetic analyses of ABC transporters from *Drosophila*, *Anopheles, Bombyx* and *Daphnia.* The GLEAN models were refined on the basis of homology, EST support and sequence analysis of PCR fragments. The organization of ABC genes and frequent gene duplications in *Tribolium* impeded the correct prediction of gene models. Therefore, we manually corrected the predicted exon/intron boundaries for some GLEAN models. The subfamily assignment of *Tribolium* ABC proteins was confirmed by blastp analyses at the NCBI webserver (http://www.ncbi.nlm.nih.gov/blast). This procedure allowed unequivocal assignment of *Tribolium* ABC transporters to respective ABCA-H subfamilies. Based on our data we reassessed a previous phylogenetic analysis of the *Tribolium* ABC transporter superfamily, which was published recently in the scope of the characterization of the silkworm ABC gene superfamily [20].

### Gene expression studies by qPCR

Total RNA was isolated from pools of embryos, young larvae, penultimate instar larvae, pharate pupae, pupae and adults using the RNeasy Mini kit (Qiagen) according to the manufacturer’s recommendation (inclusive of a DNAse treatment). To evaluate expression in different body regions of penultimate larvae, total RNA was prepared from intestinal/excretory tissues and carcass. The carcass tissue was obtained by removing the head, the two last abdominal segments and the complete gut with attached Malpighian tubules (giving rise to intestinal/excretory tissues). Next, total RNA was transcribed into cDNA with the SuperScript III First-Strand Synthesis System (Invitrogen). The cDNAs from different developmental stages and from carcass and intestinal/excretory tissues were used as templates for qPCR using pairs of gene-specific primers (Additional file 1: Table S1). qPCR was performed with the iCycler iQ Realtime PCR Detection System and iQ SYBR Green Supermix following the manufacturer recommendations (Bio-Rad). The specificity of the PCRs was confirmed by melting-curve analysis at 55–95°C. Mean normalized expression was determined according to [29]. It was calculated based on the comparison of CT values of the respective target gene and the reference gene *TcRPS6* (GenBank XP_968395.1, see Additional file 1: Table S1), whose transcript levels vary only very little between different developmental stages [30]. All CT values were obtained from three independent experiments.

### dsRNA synthesis and injection into *Tribolium castaneum*

Partial cDNAs were amplified for ABCA-H transporters using templates from appropriate developmental stages (as determined by our gene expression study) and gene-specific primers (Additional file 1: Table S1). The PCR products were ligated into the pGEM-T vector (Promega), and the resulting plasmids were used to transform competent *E. coli* DH5α cells. After culturing the bacteria in selective LB medium, the pGEM-T plasmids containing ABC-cDNA fragments were isolated using the Plasmid Mini Kit (Qiagen) and the inserts were sequenced (Seqlab GmbH, Göttingen). The sequences were aligned with the NCBI refseq mRNAs (http://blast.ncbi.nlm.nih.gov), with transcripts deduced from GLEAN models or manually curated mRNAs from the BeetleBase genome browser (http://BeetleBase.org). Synthesis of dsRNA was performed as described previously [31]. In brief, the pGEM-T vectors with the *TcABC*-cDNA inserts were used as templates in a PCR with primers containing T7 promoter sequences at the 5’-ends and sequences specific for the corresponding *TcABC*-cDNA (Additional file 1: Table S1). After agarose gel electrophoresis, the PCR products were excised and purified with the QIAquick Gel Extraction Kit (Qiagen), and subsequently used as templates for dsRNA synthesis performed with the AmpliScribe T7-Flash transcription kit (Epicentre).

RNAi experiments were performed as follows: at least 40 penultimate instar larvae, pre-pupae or young adults were ether or cold-anesthetized and injected with 200 nl of the respective *Tribolium* ABC-dsRNA (1μg/μl in 0.1 mM potassium phosphate buffer containing 5 mM KCl, pH 7.0). After injection, the insects were kept under standard conditions for visual monitoring of phenotypes and further analysis. Phenotypes were monitored on a daily basis with the help of a stereomicroscope (Zeiss Discovery V.8). Buffer containing dsRNA for the *Tribolium* tryptophan oxygenase encoding gene *vermilion* was injected as a control (*TcVer*; GenBank NP_001034499.1). To monitor the knock-down of transcript levels, total RNA was prepared from pools of three insects three days after injection of dsRNA by using the RNeasy Mini Kit (Qiagen). cDNA synthesis and qPCR experiments with gene-specific primers (Additional file 1: Table S1) were performed as described above.

### Cryosectioning and histological staining

Fixation and cryosectioning of *T. castaneum* larvae were performed using a modified protocol published previously [32]. In brief, decapitated late larvae were fixed overnight at 4°C in PBS buffer (20 mM KH_2_PO_4_, 20 mM Na_2_HPO_4_, 150 mM NaCl, pH 7.4) containing 4% (w/v) paraformaldehyde. Cryoprotectioning, tissue embedding and serial cryosectioning were performed as described previously using a Leica cryostat (CM1850, 160 mm steel blade with C profile) at −24°C and SuperFrost Plus microscope slides to collect the sections. The specimens were dried at 40°C on a hotplate for tissue adherence. After blocking for 30 min at RT with PBS buffer containing 2% (w/v) bovine serum albumin, the cryosections were stained for 60 min at RT with Nile Red (0.01 mg/ml in DMSO) or Calcofluor white (0.01% (w/v) in PBS buffer). The specimen were rinsed three times for 5 min with PBS, covered by Vectashield mounting medium (Vector Labs Inc.) and viewed with an Olympus IX70 fluorescence microscope using appropriate filter sets.

## Results

### Identification of genes encoding proteins of ABC transporter superfamily from *T. castaneum*

**Figure 1:**
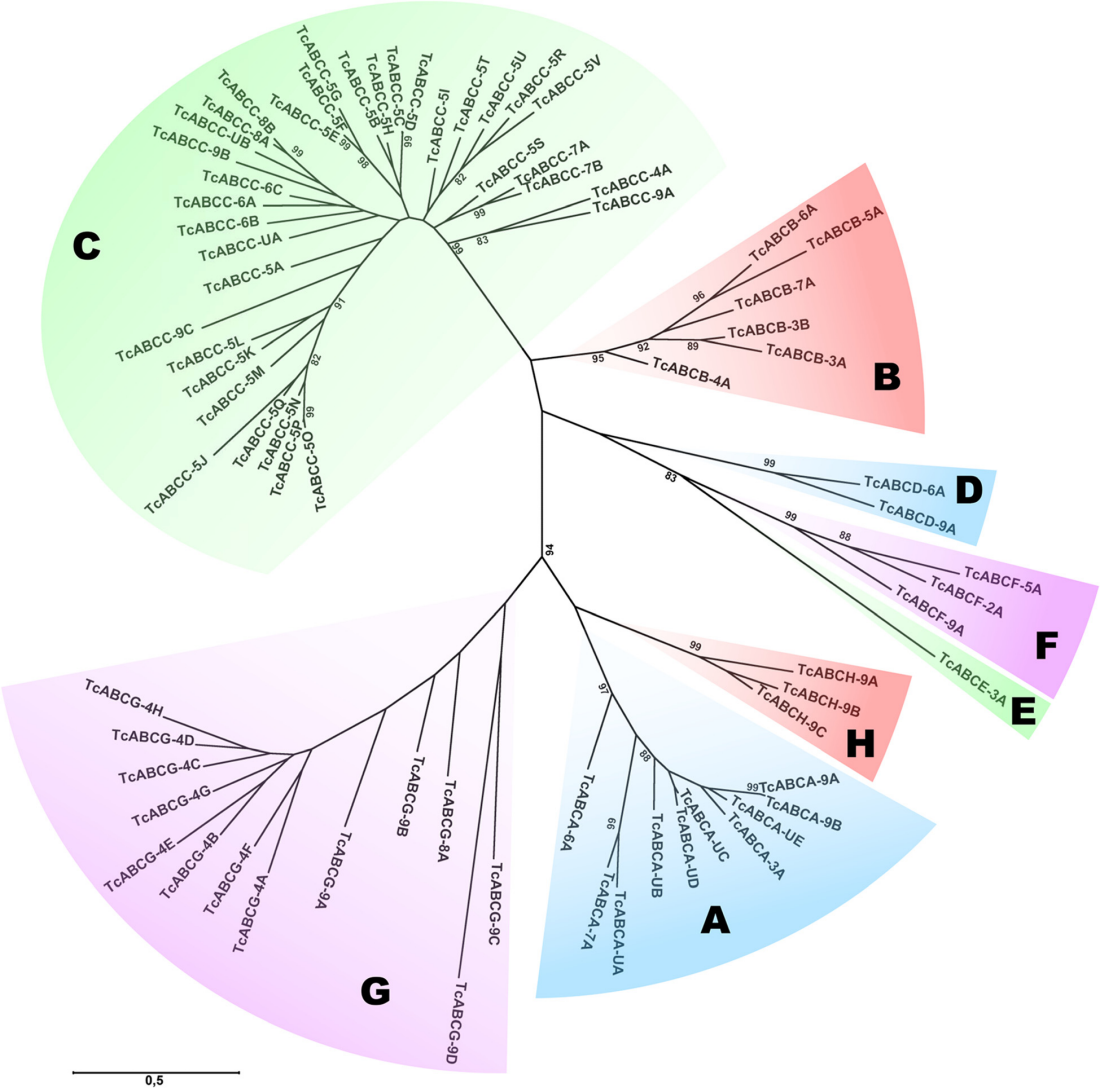
**Phylogenetic tree for *T. castaneum *ABC transporters.** The maximum likelihood tree was calculated on the basis of a ClustalW alignment (Blosum62) of nucleotide binding domains from different ABC transporters of *T. castaneum*. The scale bar indicates an evolutionary distance of 0.5 amino acid substitutions per site. Bootstrap values are given in percentages at the internodes. Different ABC transporter subfamilies are indicated by colored shadings.

To identify ABC transporters from *T. castaneum*, we performed tblastn searches using the highly conserved nucleotide-binding domains from different insect sources as a query. All putative ABC transporters with E-values lower than 10^-6^ were reanalyzed based on the architecture of their conserved domains (TMDs and NBDs). A total of 73 putative ABC transporters genes were found on eight of *T. castaneum’s* 10 linkage groups (chromosomes 2–9, see Table 1). This is five more than that reported in a previous study, where the *T. castaneum* genome was analyzed in the scope of the identification of genes encoding ABC transporters from *B. mori*[20]. Next, we performed a ClustalW multiple sequence alignment of the NBDs, and constructed a maximum-likelihood tree. Phylogenetic analysis revealed that the *T. castaneum* ABC transporters group into 8 subfamilies (A-H) with bootstrap values ≥ 95% at most of the respective branches (Figure 1).

**Table 1:** **Overview on 73 *TcABC *genes identified in the *T. castaneum *genome**

**Gene**	**REFSEQ**	**GLEAN**	**Position (Strand)**	**Mw [kDa]**	**ESTs**	**PCR verified**
**A**						
*TcABCA-UA*	XP_967122.2	10722	N/A	114.4	0	N/D
*TcABCA-UB*	XP_970623.2	02280	N/A	185.2	3	+
*TcABCA-UC*	XP_001809119.1	02278	N/A	164.2	0	+
*TcABCA-UD*	XP_970754.2	N/A	N/A	197.7	2	N/D
*TcABCA-UE*	XP_970882.2	02274	N/A	214.7	8	+
*TcABCA-3A*	XP_967691.2	10527	17,798,192.17,808,653 (+)	148.0	0	+
*TcABCA-6A*	XP_001812136.1	15859	12,187,194.12,207,176 (+)	184.6	2	+
*TcABCA-7A*	XP_969271.1	N/A	650,182.660,292 (+)	186.3	11	+
*TcABCA-9A*	N/A	16282	13,853,789.13,858,783 (−)	136.7	0	+
*TcABCA-9B*	N/A	16281	13,861,970.13,866,823 (−)	104.4	1	+
**B**						
*TcABCB-3A*	XP_967244.2	03402	5,939,075.5,943,546 (+)	131.0	2	+
*TcABCB-3B*	XP_001810982.1	03797	22,077,333.22,099,171 (+)	139.9	3	+
*TcABCB-4A*	XP_966724.1	08383	10,890,934.10,895,745 (+)	70.9	3	+
*TcABCB-5A*	XP_001813375.1	14809	19,030,456.19,033,317 (+)	78.5	2	+
*TcABCB-6A*	XP_974441.2	15192	2,726,918.2,738,164 (−)	95.1	4	+
*TcABCB-7A*	XP_972133.1	09730	6,893,504.6,896,933 (+)	76.3	2	+
**C**						
*TcABCC-UA*	XP_971058.2	08570	N/A	118.4	0	+
*TcABCC-UB*	XP_970354.2	01803	N/A	162.6	0	+
*TcABCC-4A*	XP_972214.2	08035	4,956,628.4,983,422 (+)	185.8	2	+
*TcABCC-5A*	XP_973658.1	13752	5,966,306.5,973,951 (−)	127.2	3	+
*TcABCC-5B*	XP_973693.1	13751	5,978,733.5,988,109 (−)	143.9	0	+
*TcABCC-5C*	XP_973725.2	13750	5,989,614.5,994,073 (−)	142.5	3	+
*TcABCC-5D*	XP_973757.2	13749	5,995,524.5,999,966 (−)	142.9	3	+
*TcABCC-5E*	XP_968524.2	14089	6,969,497.6,973,765 (+)	144.3	0	+
*TcABCC-5F*	XP_968603.2	14090	6,975,148.6,979,220 (+)	125.7	4	+
*TcABCC-5G*	N/A	14091	6,981,397.6,985,546 (+)	108.0	0	+
*TcABCC-5H*	XP_968748.1	14092	6,989,375.6,993,654 (+)	147.3	6	+
*TcABCC-5I*	N/A	10434	8,860,574.8,868,920 (−)	147.4	10	+
*TcABCC-5J*	XP_971732.1	14379	12,075,643.12,079,703 (+)	141.1	0	+
*TcABCC-5K*	XP_971687.2	14380	12,080,534.12,084,545 (+)	136.1	0	+
*TcABCC-5L*	N/A	14381	12,085,585.12,089,465 (+)	132.8	0	+
*TcABCC-5M*	N/A	14382	12,090,049.12,093,099 (+)	103.2	0	+
*TcABCC-5N*	XP_971802.2	14383	12,094,316.12,098,575 (+)	143.1	3	+
*TcABCC-5O*	XP_971857.2	14384	12,099,129.12,103,236 (+)	140.7	0	+
*TcABCC-5P*	XP_971908.1	14385	12,103,832.12,107,957 (+)	143.6	0	+
*TcABCC-5Q*	XP_971965.2	14386	12,109,821.12,114,012 (+)	143.3	5	+
*TcABCC-5R*	N/A	14403	12,560,442.12,565,573 (+)	150.6	6	+
*TcABCC-5S*	XP_970316.2	14589	15,369,140.15,373,635 (+)	149.6	0	+
*TcABCC-5T*	XP_969781.1	14775	18,589,675.18,598,980 (+)	146.7	5	+
*TcABCC-5U*	XP_969849.1	N/A	18,599,335.18,606,763 (+)	145.4	2	+
*TcABCC-5V*	XP_001810350.1	N/A	18,607,427.18,612,030 (+)	147.2	0	+
*TcABCC-6A*	XP_969711.1	15346	188,365.195,414 (−)	140.4	3	+
*TcABCC-6B*	N/A	(former 15131)	4,354,979.4,364,295 (−)	139.0	0	+
*TcABCC-6C*	XP_970526.1	14880	11,705,126.11,709,007 (−)	136.4	0	+
*TcABCC-7A*	XP_972486.2	09891	13,193,223.13,202,867 (+)	147.9	0	+
*TcABCC-7B*	XP_972534.1	09892	13,205,046.13,210,240 (+)	146.6	2	+
*TcABCC-8A*	XP_969997.2	06467	12,119,015.12,122,960 (+)	130.5	0	+
*TcABCC-8B*	XP_970068.2	06468	12,123,192.12,127,192 (+)	132.4	0	+
*TcABCC-9A*	XP_969354.2	12253	837,310.846,332 (+)	202.6	5	+
*TcABCC-9B*	XP_969737.2	10962	5,070,045.5,076,707 (−)	94.5	0	+
*TcABCC-9C*	XP_001813826.1	11800	18,102,317.18,105,944 (−)	68.1	0	+
**D**						
*TcABCD-6A*	XP_971218.1	15333	332,622.337,550 (−)	82.3	4	+
*TcABCD-9A*	XP_971649.2	12277	2,166,813.2,174,693 (+)	N/A	1	+
**E**						
*TcABCE-3A*	XP_968009.1	10519	17,851,529.17,857,456 (−)	68.6	21	+
**F**						
*TcABCF-2A*	XP_971562.1	04420	6,966,233.6,971,293 (−)	91.1	2	+
*TcABCF-5A*	XP_966990.1	13884	2,974,669.2,984,045 (−)	70.4	4	+
*TcABCF-9A*	XP_972814.1	11927	15,974,910.15,978,068 (−)	79.4	3	+
**G**						
*TcABCG-4A*	XP_971210.2	07470	4,859,967.4,868,694 (−)	71.3	18	+
*TcABCG-4B*	XP_971681.1	07467	4,894,214.4,898,836 (−)	70.9	6	+
*TcABCG-4C*	XP_001813184.1	07466	4,901,619.4,910,932 (−)	75.5	26	+
*TcABCG-4D*	XP_973458.1	08293	9,112,640.9,117,930 (+)	79.2	4	+
*TcABCG-4E*	XP_001811847.1	N/A	11,388,853.11,411,657 (+)	71.9	2	+
*TcABCG-4F*	XP_971735.1	07074	11,389,147.11,395,881 (−)	67.8	13	+
*TcABCG-4G*	XP_973493.1	08454	12,061,217.12,066,316 (+)	70.6	4	+
*TcABCG-4H*	XP_973526.1	07047	12,069,092.12,077,377 (−)	74.1	6	+
*TcABCG-8A*	XP_975214.2	05701	10,510,228.10,520,727 (−)	74.3	0	+
*TcABCG-9A*	XP_968696.1	11998	12,953,115.12,965,075 (−)	72.2	3	+
*TcABCG-9B*	NP_001034521.1	11997	12,969,020.12,977,333 (−)	74.4	0	+
*TcABCG-9C*	XP_968472.1	11713	19,576,197.19,580,771 (−)	102.8	1	+
*TcABCG-9D*	XP_968555.1	12778	19,586,463.19,590,663 (+)	81.5	0	+
**H**						
*TcABCH-9A*	XP_973444.1	12169	2,432,030.2,439,299 (−)	79.6	2	+
*TcABCH-9B*	XP_967359.1	12512	15,160,638.15,174,277 (+)	84.1	2	+
*TcABCH-9C*	XP_974932.1	11755	18,625,705.18,648,891 (−)	87.7	6	+

Subfamily assignment was re-evaluated on the basis of the known architecture of conserved domains. Proteins were named in accordance with the guidelines set forth by the human genome organization gene nomenclature committee (HGNC), with *Tc* indicating the species *T. castaneum*, ABC the protein superfamily, A-H the subfamily, 2–9 the chromosome (U stands for unassigned), and A-V the arrangement on the chromosome. Thus, *TcABCG-8A* is the first subfamily G ABC transporter on chromosome 8 of *T. castaneum*. We identified 10, 6, 35, 2, 1, 3, 13 and 3 ABC transporters belonging to subfamilies TcABCA, -B, -C, -D, -E, -F, -G and -H, respectively (see Table 1). In comparison to the study published by Liu et al. [20], we identified an additional TcABCA, and four additional TcABCC proteins (see Additional file 2: Table S2). Subfamilies ABCA and ABCC comprise only full-transporters. Subfamily ABCB comprises two full and four half-transporters (with TMD-NBD arrangement). Subfamilies ABCD, -G and -H contain only half-transporters, with the expected TMD-NBD arrangement for ABCD, and NBD-TMD arrangements for ABCG and ABCH. Subfamily ABCE and -F proteins contain two NBDs but, as expected, no TMDs. The number of ABC transporters within each family and their overall domain architectures were, with a few exceptions, similar to those reported for other insect species including *B. mori*, *Anopheles gambiae, Apis mellifera* and *D. melanogaster* (see Additional file 2: Table S2). The most striking observation is that subfamily ABCC has expanded significantly in the *T. castaneum* genome. This expansion is due to gene duplication events on chromosome 5, and results in *T. castaneum* having about twice as many *ABCC* genes as other sequenced insects (see Figure 1).

### RNAi screen to identify ABC genes with crucial functions in the development of *T. castaneum*

**Figure 2:**
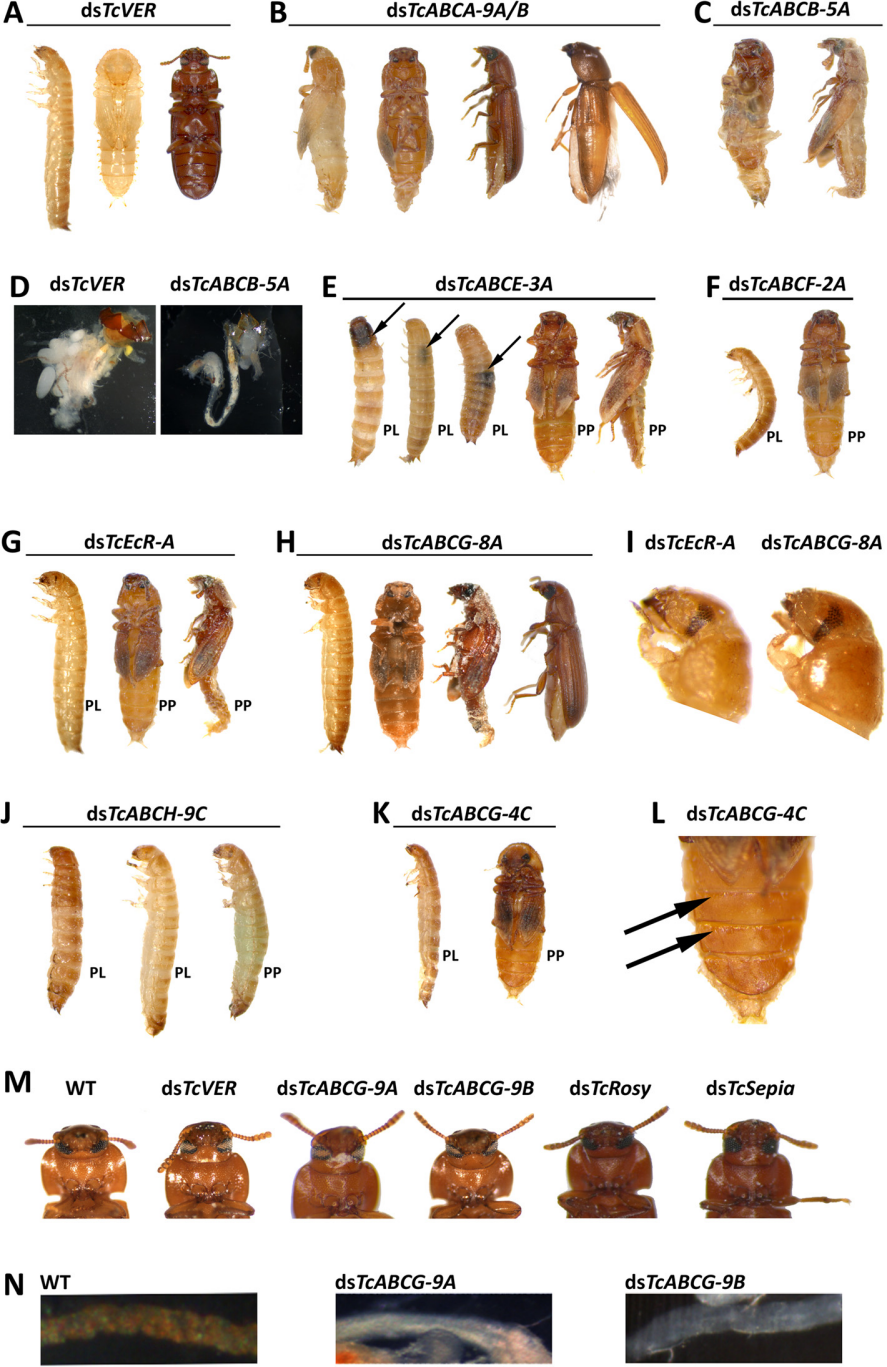
**Effects of dsRNA-injections on the development of *T. castaneum*.** dsRNAs specific for different *TcABC*, *TcEcR-A* and *TcVer* genes were injected into penultimate instar larvae (PL), pre-pupae (PP), or adults (AD), and the phenotypes were monitored. (**A**) *TcVer* - injection into PL or PP (PLI or PPI): normal development, except for the expected white-eye phenotype. (**B**) *TcABCA-9A/B* - PLI: wing defects, elytral shortening and blisters. (**C**) *TcABCB-5A* - PLI or PPI: severe molting defects during pupal-adult molt. (**D**) *TcABCB-5A* – injection into AD (ADI): disruption of ovary maturation and egg development. (**E**) *TcABCE-3A* - PLI: larval growth arrest and death before molting. Note, localized zones of melanization (arrows). PPI: pharate pupae died before adult eclosion. (**F**) *TcABCF-2A* - PLI: death in the quiescent stage. PPI: growth arrest and death as pharate adults. (**G**) *TcEcR-A* - PLI: death in the quiescent stage. PPI: pupal-adult intermediates, defects in wing development. (**H**) *TcABCG-8A* – PLI and PPI: molting defects and developmental arrest; adult beetles with shortened elytra. (**I**) *TcEcR-A* or *TcABCG-8A* - PLI: prematurely developed compound eyes. (**J**) *TcABCH-9C* - PLI: death during the quiescent stage before molting. PPI: abortive larval-pupal molt and death as pharate pupae. (**K**) *TcABCG-4C* - PLI: death during the quiescent stage before molting; PPI: death at the pupal stage due to desiccation. (**L**) *TcABCG-4C* - PPI: detail view on the ventral abdomen of a pupa with traces of water bleeding at the intersegments (arrows). (**M**) *TcVer*, *TcABCG-9A* and *TcABCG-9B* - PLI: white-eye phenotype. *TcRosy* and *TcSepia -* PLI: wild-type eye and cuticle colorings. (**N**) *TcABCG-9A* and *TcABCG-9B* - PLI: white Malpighian tubules in adults (reddish-brown Malpighian tubules in wild-type beetles).

To examine the role of individual ABC transporters in the development of *T. castaneum*, we synthesized dsRNA for all *TcABC* genes from subfamilies A-H and injected them into penultimate larvae. As a mock control, we injected *TcVer*-dsRNA to knock-down expression of the *tryptophan oxygenase* gene, *vermilion*, leading to a white-eye phenotype (see Figure 2A, [33]). Knock-down of the respective mRNA was monitored by qPCR. The effects of dsRNA injections on development and viability were monitored on a daily basis until adult eclosion and beyond. In this screen, we found that 10 of 73 *TcABC*-dsRNA injections resulted in developmental phenotypes. To confirm these phenotypes, we used a second, non-overlapping dsRNA for larval injection, and in all cases the injection led to the same phenotype. The genes for which RNAi led to developmental phenotypes encoded subfamily A transporters TcABCA-9A (XP_966841.2) and TcABCA-9B (XP_966932.2), subfamily B transporter TcABCB-5A (XP_001813375.1), subfamily E protein TcABCE-3A (XP_968009.1), subfamily F protein TcABCF-2A (XP_971562.1), subfamily G transporters TcABCG-4C (XP_001813184.1), TcABCG-8A (XP_975214.2), TcABCG-9A (XP_968696.1), TcABCG-9B (NP_001034521.1) and subfamily H transporter TcABCH-9C (XP_974932.1). qPCR revealed that in every case transcript levels were significantly silenced after dsRNA injection in penultimate larvae, pre-pupae or adults (Additional file 3: Figure S1).

All other tested *TcABC* genes did not reveal detectable phenotypes when dsRNA was injected into penultimate larvae. Absence of phenotypes may be explained either by redundant substrate specificities (particularly for subfamily C transporters), non-essential functions, or specific functions at developmental stages that were not assessed by larval injections. We can also not completely rule out the possibility that we might have missed some phenotypes due to insufficient silencing of the target genes. However, in the case of TcABCC-9A (XP_969354.2) and its closest paralogue TcABCC-4A (XP_972214.2, see Figure 1) we monitored the absence of phenotypes very carefully by multiple injections. TcABCC-9A is the closest homolog of the *D. melanogaster* SUR, which has been suggested to be a target for diflubenzuron and gets inhibited upon binding of diflubenzuron resulting in a defect in Ca^2+^ signaling and, as a consequence, in a block of cuticle secretion [18]. Correspondingly, we expected to see abortive moltings and defects in egg hatching due to inhibition of chitin synthesis, as we have recently reported it for diflubenzuron-treated larvae of *T. castaneum*[34]. However, we obtained no phenotypes for TcABCC-9A or its closest paralogue TcABCC-4A even at high dsRNA concentration and with dsRNAs for different gene regions. To prevent complementation due to the possibility of overlapping functions, we silenced both genes simultaneously by co-injection of the corresponding dsRNAs. However, even knocking-down both transcripts at the same time failed to reveal molting or egg-hatching defects. Injection of dsRNA for three other ABC transporters grouping next to TcABCC-9A/C4A also failed to reveal phenotypes (see Figure 1). In summary, our data suggest that SUR homologs do not play pivotal roles in cuticle formation and chitin synthesis in *T. castaneum* under normal physiological conditions. However, we cannot exclude the possibility that SURs may have essential functions under xenobiotic stress.

### Characterization of phenotypes caused by RNAi for *TcABC* genes

The phenotypes caused by silencing of ten *TcABC* genes were further investigated. The main phenotypic characteristics and mortality rates for these are summarized in Table 2. The spectrum of phenotypes included growth arrest and localized melanization, eye-pigmentation defects, abnormal cuticle formation, egg-laying and egg-hatching defects and mortality due to abortive molting and desiccation. If the phenotype was lethal, mortality ranged between 30% and 100% depending on which gene was silenced.

**Table 2:** **Summary on phenotypes caused by RNAi for *TcABC *genes**

**Gene**	**Mortality**	**Mortality**	**Phenotype**	**Phenotype**	**Phenotype**
	Injection into larvae	Injection into pre-pupae	Larvae/Pharate pupae	Pupae/Pharate adults	Adults
*TcABCA-9A*	N/A	30%	N/A	Wing defects, elytral shortening	Wing defects, elytral shortening and blisters
*TcABCA-9B*	N/A	30%	N/A	Wing defects, elytral shortening	Wing defects, elytral shortening and blisters
*TcABCB-5A*	100%	50%	N/A	Severe molting and developmental defects during pupal-adult molt	Female adults do not produce eggs
*TcABCE-3A*	100%	100%	Arrest in quiescent stage, localized melanization, very small	Arrest as pharate adults, no adult eclosion	N/A
*TcABCF-2A*	100%	100%	Arrest in quiescent stage, very small	Arrest as pharate adults, no adult eclosion	N/A
*TcABCG-8A*	50%	50%	Arrest during molting, compound eye already in larvae	Wing defects, elytral shortening, dorsal abdomenexposed	Elytral shortening
*TcABCG-9A*	0%	0%	No phenotype	White eyes	White eyes and white Malpighian tubules
*TcABCG-9B*	0%	0%	No phenotype	White eyes	White eyes and white Malpighian tubules
*TcABCG-4C*	100%	100%	Arrest in quiescent stage, rough cuticle, due to shrinkage and desiccation	Arrest in the pupal stage, death before molting	Significant reduction in the number of eggs laid, no egg-hatching
*TcABCH-9C*	100%	100%	Arrest in quiescent stage, rough cuticle, due to shrinkage and desiccation	abortive larval-pupal molting	Significant reduction in the number of eggs laid, no egg-hatching

#### TcABCA-9A/B

**Figure 3:**
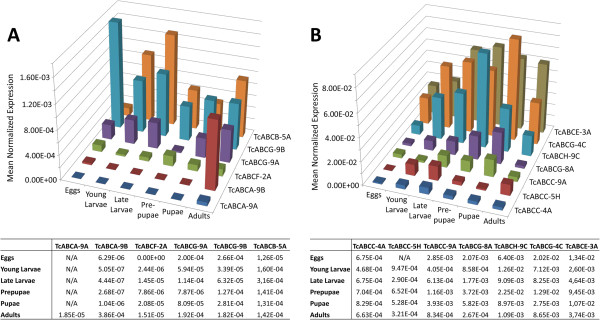
**Expression profiles for *TcABC *genes at different developmental stages as determined by qPCR.** Total RNA was isolated from pools of eggs, young larvae, late larvae, pre-pupae, pupae and adults and transcribed into cDNA. Triplicates of independent cDNA preparations were used as templates in qPCRs with primers specific to the respective genes. Mean normalized expression was determined by comparing CT values of the respective target gene and the reference gene *TcRPS6*. Standard errors are given in the tables below. Panel (**A**) shows the expression profiles for *TcABC* genes with comparably low transcript levels, whereas panel (**B**) shows that for *TcABC* genes with higher transcript levels.

Subfamily A transporters in mammals have been reported to play important functions in cell physiology as they control lipid transport [35]. To date, no specific function has been reported for ABCA transporters in insects. However, our RNAi screen revealed phenotypes for two of the ten ABCA transporters tested. Injection of dsRNA specific for *TcABCA-9A* or *TcABCA-9B* resulted in ~30% mortality. Lethality was stage specific, as it occurred during or after adult eclosion even though dsRNA was injected into larvae. The pupae and pharate adults showed severe defects in the development of wings and elytra. Due to elytral shortening the dorsal abdomen remained partially uncovered (Figure 2B). Strikingly, adults developed liquid-filled blisters on their elytra. These blisters started to form during late pupal development and were bilateral or unilateral (if unilateral there was no site preference). In addition, the elytral cuticle appeared irregular and rough compared to control beetles (data not shown). In line with the stage specificity of the observed phenotypes, *TcABCA-9A* and *TcABCA-9B* transcripts were nearly undetectable in all but at adult stages (see also Figure 3A). Interestingly, the genes share a high degree of sequence identity and are adjacent to one another on chromosome 9, suggesting that they evolved from a recent gene duplication event (Additional file 4: Figure S2). As a consequence, we were unable to synthesize dsRNA capable of discriminating between the two genes. However, since *TcABCA-9A* exhibits only very weak expression (Figure 3A), we conclude that the observed phenotype is mainly (or perhaps solely) due to silencing of *TcABCA-9B*.

#### *TcABCB-*5A

In human, several subfamily B transporters have been reported to mediate multiple drug resistance of cancer cells, and in insects they seem to be involved in insecticide resistance [36,37]. Our RNAi screen revealed phenotypic alterations for only one of the six *TcABCB* genes. Specifically, injection of *TcABCB-5A-*dsRNA into penultimate larvae or pre-pupae caused severe developmental defects which manifested during the pupal-adult molt and caused lethality. The pupae showed strong morphological abnormalities after pre-pupal injection and were partially trapped in the pupal cuticle (Figure 2C). To evaluate the effects on egg development, we injected dsRNA for *TcABCB-5A* into adult females, mated them with wild-type males and analyzed egg-laying. While ds*TcVer*-injected females reproduced normally, ds*TcABCB-5A*-injected females were sterile because their ovaries failed to produce eggs, possibly due to starvation (Figure 2D).

#### TcABCE-3A and TcABCF-2A

Subfamily E and F proteins are highly conserved across all phyla, and thus must have also important functions in insects. They lack TMDs and therefore do not function as transporters. Instead they play a role in ribosomal assembly, translational control and mRNA transport. Injection of dsRNA specific for *TcABCE-3A*, the homolog of the *Drosophila pixie* gene [38], into penultimate larvae resulted in a lethal phenotype with 100% mortality. Injected larvae arrested during the quiescence stage, were smaller than control larvae and developed localized melanization zones at different body regions, which were distant from the sites of injection (Figure 2E). Injection of dsRNA for *TcABCE-3A* into pre-pupae caused defects in pupation and led to 100% mortality. Injection of dsRNA specific for *TcABCF-2A* into penultimate larvae resulted also in a lethal phenotype with 100% mortality. Injected larvae died during the quiescent stage before the next larval molt (a stage particularly dependent on protein biosynthesis). Larvae injected with dsRNA specific for *TcABCF-2A* were signifiantly smaller than those in the control group. Injection of dsRNA specific for *TcABCF-2A* into pre-pupae caused the insects to arrest as pharate adults, which died within 4–5 days without adult eclosion (Figure 2E).

#### TcABCG-9A and TcABCG-9B

Subfamily G proteins are half-transporters and form homo- or heterodimers. Some ABCG proteins function as obligate heterodimer pairs, while others dimerize with more than one partner. This exchange of one half-transporter for another can alter substrate specificity. In mammals, ABCG proteins serve numerous physiological functions due to their ability to transport lipids, sterols, and drugs [39]. To determine the role of ABCG half-transporters in *T. castaneum*, we silenced all genes encoding putative ABCG proteins. In four cases we observed phenotypes, two of which affected eye-pigmentation (Table 2 and Figure 2M). *TcABCG-9B*, which is most closely related to the *D. melanogaster white* eye-color gene, was targeted first, because in *Drosophila*, White is required for the transport of both pteridine (red) and ommochrome (brown) pigments. Moreover, absence of White is known to result in white-eyed flies [11]. As expected, injection of dsRNA specific for *TcABCG-9B* resulted in a white-eye phenotype (Figure 2M).

In *Drosophila*, White is thought to dimerize with the ABCG proteins Scarlet and Brown, which transport ommochrome and pteridine pigments, respectively. As suggested by the name, loss of functional Scarlet results in red-eyed flies, while loss of functional Brown results in brown-eyed flies [11]. Interestingly, injection of dsRNA specific for *TcABCG-9A*, which is most closely related to the *D. melanogaster scarlet* gene, produced a white-eye phenotype indistinguishable from that of *TcABCG-9B* knock-down (Figure 2M).

The absence of reddish pteridine pigments could possibly be explained by the lack of a functional Brown ortholog, or by the absence of the biosynthetic pathway for pteridine-based pigments. To test these possibilities we silenced the closest *T. castaneum* homologs of the *D. melanogaster* genes *sepia* and *rosy*, both encoding enzymes involved in pteridine biosynthesis. Neither resulted in a discernible phenotype (Figure 2M), in particular no alterations in eye or cuticle coloring. Like White and Scarlet in *Drosophila*, TcABCG-9A and TcABCG-9B play functional roles in the Malpighian tubules of *T. castaneum*. This is not surprising since this is where tryptophan metabolites are processed. Accordingly, injection of dsRNA for either *TcABCG-9A* or *TcABCG-9B* resulted in the loss of reddish-brown pigments normally observed in the Malpighian tubules of wild-type beetles. After RNAi-mediated knock-down of these transcripts we could detect only white Malpighian tubules (Figure 2N).

#### TcABCG-8A

Injection of dsRNA specific for another subfamily G member, *TcABCG-8A*, resulted in molting defects, developmental arrest and a mortality rate of about 50% (Table 2). When *TcABCG-8A*-specific dsRNA was injected into larvae, the insects failed to molt (Figure 2H). Moreover, they prematurely developed compound eyes. Normally the compound eyes develop during the pupal stage, but in *TcABCG-8A*-dsRNA injected larvae the compound eyes could be seen under the old cuticle (Figure 2I). Approximately 50% of the pre-pupae injected with *TcABCG-8A*-dsRNA failed to develop into adults and eventually died. However, if adult eclosion was successful, the beetles exhibited abnormal development of elytra and wings, and failed to cover the dorsal side of the abdomen (Figure 2H).

The observed phenotype resembles that reported for RNAi-mediated knock-down of the *T. castaneum* ecdysone receptors TcECR-A and TcECR-B [40], which also cause larval arrest, abnormal development of elytra and wings during pupation, and abnormal compound eye development (shown for *TcECR-A* in Figures 2G, I). This finding suggests that TcABCG-8A and TcECR-A/B function in the same developmental pathway.

#### TcABCG-4C and TcABCH-9C

Injection of dsRNA specific for a subfamily G member, *TcABCG-4C*, and a subfamily H member, *TcABCH-9C*, caused similar phenotypes. Despite differences, injections resulted in desiccation and 100% mortality (Table 2). Injection of either dsRNA into young larvae arrested development and rapidly caused death during the quiescent stage. Injected larvae exhibited a rough cuticle as a consequence of desiccation and shrinkage (Figures 2J and K). While injection of dsRNA specific for *TcABCH-9C* into pre-pupae resulted in abortive larval-pupal molting and death as pharate pupae (Figure 2J), injection of *TcABCG-4C*-dsRNA caused death at the pupal stage before the pupal-adult molt (Figure 2K). In both cases death was a result of desiccation. Specifically, we detected traces of fluid bleeding at the intersegmental cuticle of affected pupae (Figure 2L), and injected insects never reached the adult stage.

**Figure 4:**
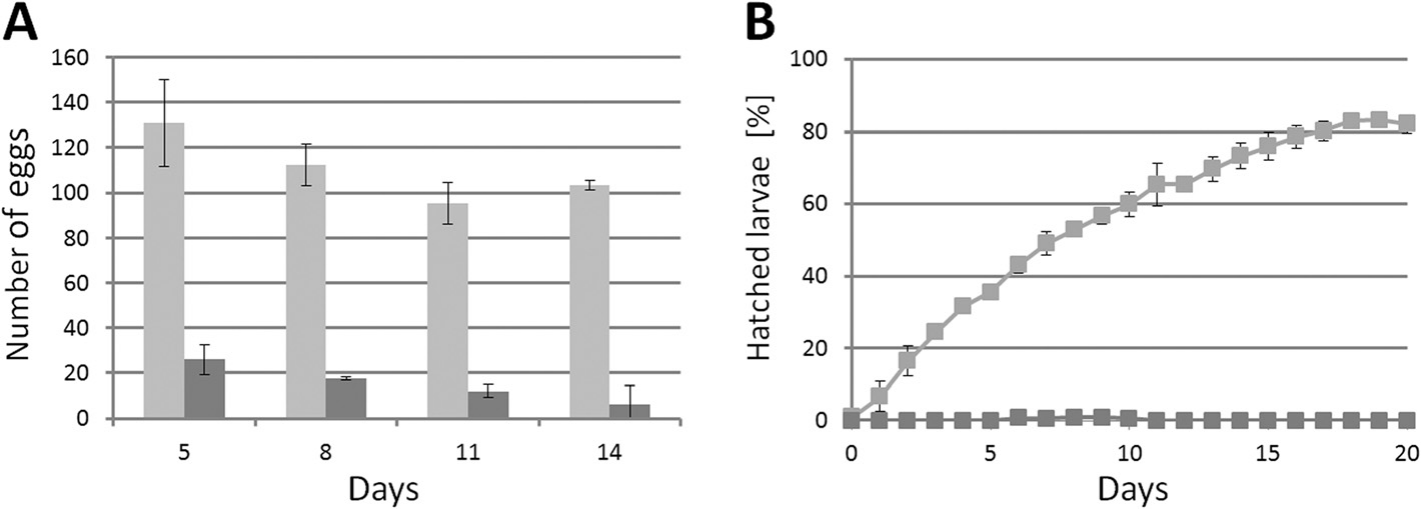
**The effect dsRNA injection specific for *TcABCG-4C *on egg-laying and egg-hatching.** dsRNA specific for *TcVer* (light bars) or *TcABCG-4C* (dark bars) was injected into 20 young female adults, which were then mated with wild-type males five days after injection. (**A**) The eggs laid within a period of two weeks were counted every third day, starting at day five after mating. (**B**) The number of larvae that hatched from the eggs was quantified. The percentage of hatched larvae was calculated relative to the number of laid eggs. Means ± S.E. (n = 3) are shown.

**Figure 5:**
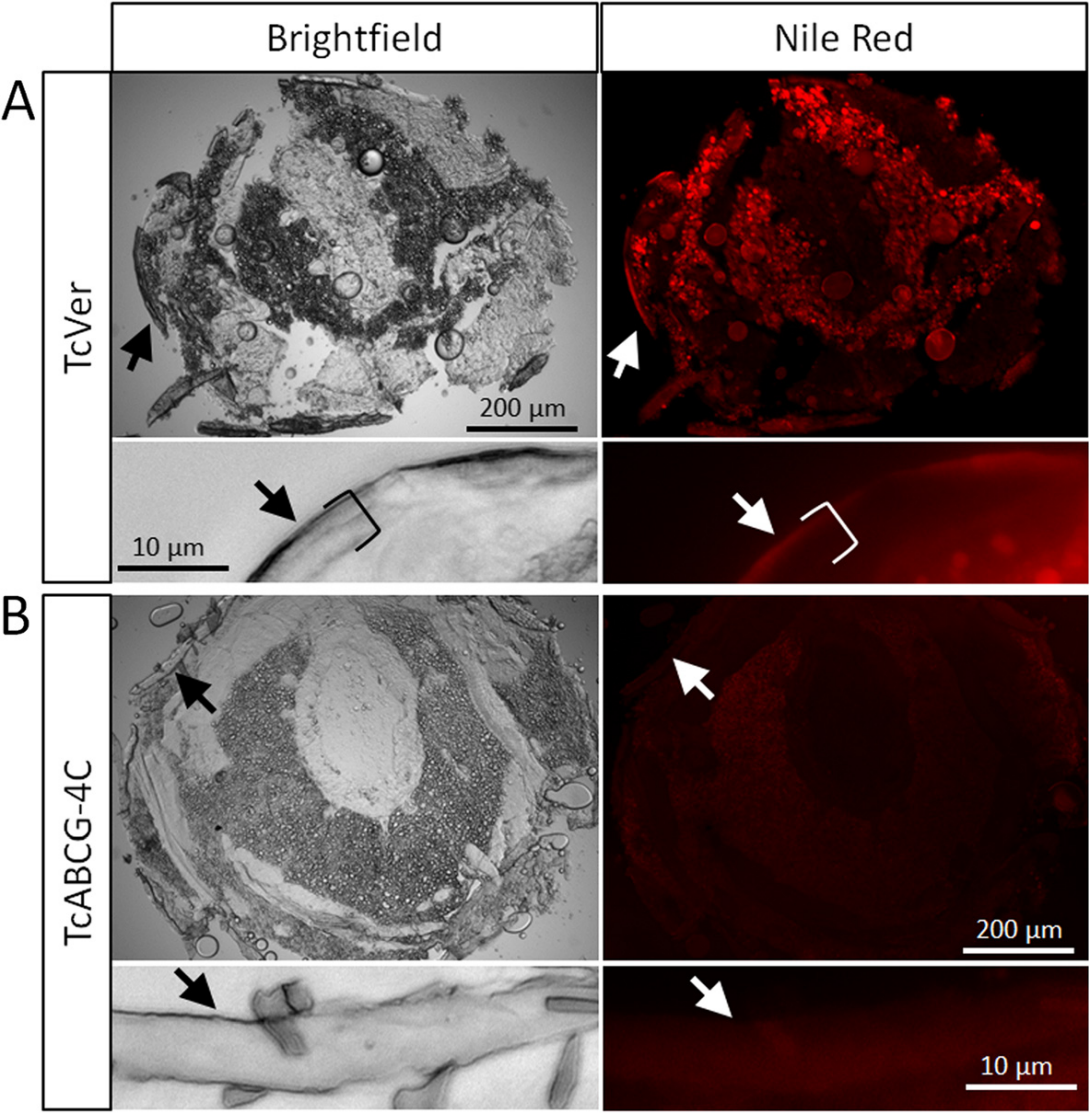
**Nile red staining of cryosections from *T. castaneum *late larvae injected with dsRNA specific for *TcVer *and *TcABCG-4C.*** To detect neutral lipids such as triglycerides, cryosections (transverse, 20 μm) prepared from the abdomen of late larvae were stained with Nile red. (**A**) dsRNA specific for *TcVer* was injected into penultimate larvae. The upper *right panel* shows a strong fluorescence labeling of fat body cells, fat droplets and cuticle (arrow) at low magnification; *left panel*, bright field micrograph. The lower panels show detail views of the cuticle in bright field (left) and fluorescence imaging (right). Nile red stains cuticular lipids of the epicuticle. The thickness of the cuticle is marked with a bracket. (**B**) dsRNA specific for *TcABCG-4C* was injected into penultimate larvae. The *right panel* shows only very weak fluorescence labeling of fat body cells and fat droplets, and no staining of the cuticle (arrow); *left panel*, bright field micrograph. The lower panels show detail views of the cuticle in bright field (left) and fluorescence imaging (right). Nile red did not stain the epicuticle.

To determine the effects of gene knock-down on egg-laying and egg-development, we injected dsRNA into adults, then counted and inspected their eggs every three days. Injection of either *TcABCG-4C*- or *TcABCH-9C*-specific dsRNA drastically reduced the number of eggs laid, and all eggs failed to hatch (shown exemplarily for *TcABCG-4C* in Figure 4). The observed phenotypes suggest that TcABCG-4C and TcABCH-9C act as transporters of cuticular lipids, which are deposited in the outer epicuticular layer to prevent water-loss. To examine the effects of RNAi for *TcABCG-4C* on lipid content and distribution, we treated cryosections of ds*TcVer* and ds*TcABCG-4C*-injected larvae with Nile red, a fluorescent dye that stains particularly neutral lipids such as n-alkanes, triglycerides and cholesterol esters. In the ds*TcVer* injected larvae, we observed a strong fluorescent signal predominantly in the fat body but also in the epicuticle (Figure 5A). The fluorescent signal in the fat body decreased dramatically in ds*TcABCG-4C*-injected larvae indicating a depletion of triglycerides due to lipid mobilization during starvation. However, the cuticular labeling also disappeared, indicating that the epicuticle lacks lipids and consequently loses waterproofing (Figure 5B). As we obtained similar results for *TcABCH-9C*, these findings suggests that both ABC transporters are involved in the transport of neutral lipids to the cuticle and thus are required for formation of a waterproof barrier in the epicuticle.

### Expression pattern of *TcABC* genes in different developmental stages

qPCR was used to study the expression patterns of select *TcABC* genes at different developmental stages, as well as within the intestinal/excretory tissues and carcass of penultimate larvae (see Figures 3 and Additional file 5: Figure S3). Significant mRNA levels for *TcABCA-9B* were detected only in adult stages (Figure 3A). By contrast, we detected only trace amounts of *TcABCA-9A* mRNA, which were slightly more abundant in adult stages (Figure 3A). The mRNA levels for *TcABCB-5A* peaked in larval and adult stages with moderately high values, but were comparably low in eggs (Figure 3A). *TcABCB-5A* transcript levels were nearly identical in the intestinal/excretory tissues and carcass (Additional file 5: Figure S3A). Transcript levels for *TcABCF-2A* were generally very low with maximal levels in the pre-pupal stage (Figure 3A). While only low levels of *TcABCF-2A* mRNA were detected in eggs and whole larvae, the intestinal/excretory tissues and carcass of penultimate larvae possessed significantly higher levels of *TcABCF-2A* transcripts (Additional file 5: Figure S3A). Transcript levels for the eye-color genes, *TcABCG-9A* and *TcABCG-9B*, were relatively high in larval and pupal stages, and were also detectable in the intestinal/excretory tissues and carcass of penultimate larvae. Note, that intestinal/excretory tissues also contain Malpighian tubules, which appear, as in the case of *D. melanogaster*, to accumulate tryptophan-derived pigments. In pre-pupae, only relatively low transcript amounts were detected for *TcABCG-9A* and *TcABCG-9B*. Generally, *TcABCG-9B* mRNA amounts were higher than those for *TcABCG-9A* (Figure 3A). Interestingly, the highest level of *TcABCG-9B* mRNA was detected in embryos, suggesting some important function of this gene during embryogenesis. In comparison to the other tested *TcABC* genes, the expression levels appeared to be generally low for the genes discussed so far (note the scaling difference in Figures 3A and 3B). *TcABCE-3A* transcripts were highly abundant throughout all life stages with somewhat lower levels in eggs and young larvae (Figure 3B), and they were almost equally detectable in the intestinal/excretory tissues and carcass (Additional file 5: Figure S3B). The expression pattern for *TcABCG-8A*, the orthologue the *D. melanogaster E23* gene, was in line with that known for early ecdysone-responsive genes. The mRNA levels started to rise significantly in late larvae and peaked in the pupal stage. Significant expression was observed in intestinal/excretory tissues and in carcass preparations. In embryos and adults, only low transcript levels were detectable for *TcABCG-8A*. The expression profiles for *TcABCG-4C* and *TcABCH-9C* were somewhat similar (Figure 3B). In both cases transcription increased over time, going from relatively low abundance in embryos to significantly higher levels in pre-pupal and pupal stages, and then dropping back to low levels during the adult stage. In contrast to *TcABCH-9C*, the expression of *TcABCG-4C* was higher in intestinal/excretory tissues than in carcass tissues (Additional file 5: Figure S3B).

The expression profiles of three further genes were interesting for us. Of particular interest is *TcABCC-5H*, which appears to be homologous to the mammalian gene encoding the cystic fibrosis transmembrane conductance regulator (CFTR), a subfamily C protein that transports chloride and thiocyanate ions across epithelial cell membranes. qPCR showed low transcript levels in all tested developmental stages except for embryos (Figure 3B). Moreover, the finding that expression is comparably higher in intestinal/excretory tissues than in the carcass suggests that this transporter predominantly functions in midgut, or in Malpighian tubules. We also tested the expression of *TcABCC-4A* and *TcABCC-9A*, which encode homologs of *D. melanogaster* SUR. While both genes were expressed in all developmental stages and tissues tested, transcript levels were comparably low in embryos.

## Discussion

Because little is known about ABC transporter functions in insects, we examined these proteins in *T. castaneum*, a well-established and powerful genomic insect model that is highly susceptible to systemic RNAi [22]. Bioinformatic analyses revealed that the ABC transporter superfamily comprises 73 genes in *T. castaneum*, which group into eight subfamilies. With 35 members, subfamily C constitutes the largest ABC transporter subfamily in *T. castaneum.* Compared with ABCC subfamilies in other insects, TcABCC appears to have undergone a recent expansion on chromosome 5. In insects, ABC transporters have been implicated in insecticide resistance by increasing the efflux capacity for xenobiotics [37]. This suggests the possibility that the known propensity of this species to develop insecticide resistance might be related to the expansion of subfamily C genes. Indeed, extensive forms of gene duplication (or amplification) are frequently observed for genes involved in insecticide detoxification, including genes encoding esterases, glutathione-S-transferases and cytochrome P450s [41].

Our RNAi-based screen of all 73 putative *T. castaneum* ABC transporters revealed obvious phenotypes for ten genes (from subfamilies A, B, E, F, G and H). Interestingly, RNAi targeting members of the largest subfamily, ABCC, failed to reveal detectable phenotypes. This might be particularly due to gene duplication events in this subfamily giving rise to ABCC proteins with overlapping substrate specificities. A similar situation is seen in subfamily A, specifically; RNAi targeting *TcABCA-9A* and *TcABCA-9B* caused similar hypomorphic phenotypes with a mortality rate of about 30% during the pupal-adult molt. These genes are adjacent to one another on chromosome 9 and encode highly similar ABC transporters (with >80% amino acid identity in the overlapping region) which differ only slightly in the lengths of their *N*- and *C*-termini (see Additional file 4: Figure S2). *TcABCA-9A* and *-9B* appear to be paralogous genes that arose from a recent gene duplication event and may still have the ability to compensate for one another. However, since *TcABCA-9A* exhibits only low transcript levels, it is likely that the observed phenotype is due to the knock-down of *TcABCA-9B* transcripts alone.

Next to *ABCC* genes, *ABCB* and *ABCG* genes have been implicated in insecticide resistance [37]. In particular, dietary exposure of *D. melanogaster* flies to methotrexate significantly increased the expression of *ABCC* and *ABCB* genes encoding dMRP, MDR49, -50 and −65. Moreover, for the ABCB transporter MDR49 a function in germ cell migration has been suggested in transporting lipid-modified peptides that act as chemoattractants [12]. The finding that the injection of dsRNA for *TcABCB-5A*, which is highly similar to the *Drosophila* gene encoding MDR49, led to severe developmental defects during pupation and abortive pupal-adult molting, may imply that a developmental process that involves diffusible chemoattractants is impaired. Also embryogenesis could be affected, as females injected with dsRNA for *TcABCB-5A* failed to produce eggs.

Severe RNAi-induced phenotypes were observed for *TcABCE-3A* and *TcABCF-2A*. Specifically, knock-down resulted in severe developmental defects and high mortality, with larval death occurring within 4–5 days. Both genes encode highly conserved (BlastP E-values = 0.0 between beetle and human), soluble ABC proteins that lack transport functions. *TcABCE-3A* is orthologous to the human *ABCE1* gene known to encode a ribonuclease L inhibitor (RLI1). This protein plays a catalytic role in the initiation of translation, a function known to be highly conserved from yeast to human [38]. RLI1/ABCE-1 proteins also appear to play an important role in translation termination and ribosome recycling in eukaryotes. In light of RLIs’ pivotal function in protein biosynthesis, it is not surprising that the RNAi-mediated knockdown of *TcABCE-3A* in *T. castaneum* led to growth arrest and death. *ABCE-1* was also shown to inhibit the antiviral activity of interferon in humans, and hence to modulate innate immune response against viral infections including HIV [42,43]. The finding of localized melanization zones in *Tribolium* may indicate that some immune functions related to melanization are modulated by *TcABCE-3A*. Like *TcABCE-3A*, the RNAi-mediated knock-down of *TcABCF-2A* resulted in 100% mortality at the larval stage. In yeast, the orthologous gene, *ARB1*, appears to stimulate multiple steps in the 40S and 60S ribosomal biogenesis pathways [44]. Loss of this gene is lethal due to an inability to maintain protein biosynthesis. In line with the essential function of *TcABCF-2A* in *T. castaneum*, *D. melanogaster* strains carrying a recessive P-element insertion in the analogous gene, CG1703^PL16^, are homozygous lethal [45]. The finding that ABCE-3A and ABCF-2A function are essential in phylogenetically distant insect species implies that other arthropods, including plant-feeding and plant-sucking pests in agriculture and forestry, may also be susceptible to RNAi targeting *ABCE* and *ABCF* genes. Of particular importance is the observation, that RNAi caused death in the larval stage, which is frequently the most problematic stage in terms of feeding damage. Hence, it might be expected that the larval stage of a wide-range of pest species could be controlled via RNAi targeting either *TcABCE-3A* or *TcABCF-2A* orthologues. Comparable RNAi strategies generating transgenic plants producing dsRNA have been reported for coleopteran, lepidopteran or hemipteran pests [46].

RNAi targeting two *TcABCG* genes resulted in eye-color phenotypes. The best characterized ABCG transporter genes in insects are involved in the transport of eye pigment precursors. *Drosophila* mutants defective in the *white* gene encoding an ABCG half-transporter develop white eyes due to the lack of eye pigments. The ‘White’ transporter has been shown to form a heterodimer with two other ABCG half-transporters, ‘Scarlet’ and ‘Brown’. These heterodimers reside in the membranes enclosing pigment granules of specialized pigment cells within each ommatidium [47]. In *D. melanogaster* ‘White-Brown’ dimers transport pteridine precursors, which are converted into red-toned pigments, whereas ‘White-Scarlet’ dimers transport tryptophan/kynurenine-derived ommochrome precursors, which are converted to brown pigments. Correspondingly, mutants defective in the *white* gene have white eyes (i.e. complete loss of pigmentation), *brown* mutants have dark brown eyes (lack of red pigments), and *scarlet* mutants have bright red eyes (lack of brown pigments) [11]. In *T. castaneum*, the situation is obviously different, because the knock-down of *TcABCG-9A* (*scarlet*) revealed a white-eye phenotype. Despite the obvious disparity between the loss-of-function phenotypes observed for *scarlet* in *Drosophila* and *TcABCG-9A* in *Tribolium* this outcome was not unexpected. In fact, our mock controls (injection dsRNA specific for *TcVer*) predicted this result. Specifically, tryptophan oxygenase (TcVer), like White and Scarlet, is part of the ommochrome biosynthetic pathway [48,49], and as seen in our mock controls, injection of dsRNA specific for *TcVer* results in white-eyed beetles, even though only the ommochrome pathway is disrupted. Since loss of a single pigment pathway leads to a complete absence of eye pigmentation, it has been hypothesized that the *Tribolium* eye is pigmented by ommochromes alone [33,50]. It has also been noted that in *T. castaneum*, the ommochrome biosynthetic pathway produces red pigments, rather than the brown pigments observed in *Drosophila*[33]. Our data not only support this hypothesis, but also clearly demonstrate that TcABCG-9A and TcABCG-9B are part of the ommochrome pathway, likely acting as a heterodimer to transport ommochrome pigments. In addition, our database searches failed to reveal a Brown ortholog.

In *D. melanogaster*, w*hite*, *scarlet* and *brown* are also expressed in Malpighian tubules and the brain. This may explain why we detected expression of *TcABCG-9A* (*scarlet*) and *TcABCG-9B* (*white*) not only during pupation, where the adult eye is formed, but also in all other tested stages except for pre-pupae. In particular, *TcABCG-9A* and *TcABCG-9B* were also expressed in the intestinal/excretory tissues including Malpighian tubules, where these ABC transporters function in the concentration of tryptophan and purines such as guanosine [51,52]. Our observation that adult beetles injected with dsRNA for *TcABCG-9A* and *TcABCG-9B* at the pre-pupal stage have white instead of reddish-brown Malpighian tubules supports a role in the tryptophan and/or kynurenine transport. In addition, flies homozygous for mutations in either *white*, *brown*, or *scarlet* have neural phenotypes with abnormal behavior in the presence of volatile anaesthetics due to altered concentrations of biogenic amines in the brain [53]. This in turn may explain differences in susceptibilities to ether anesthesia in *T. castaneum*.

*TcABCG-8A* is an orthologue of the *D. melanogaster E23* gene, and appears to serve a similar function in *T. castaneum*, playing a role in ecdysteroid-transport [54]. This conclusion is based on the similarities in expression profiles, and on the finding that RNAi-mediated knock-down of *TcABCG-8A*, as well as *TcECR-A* and *TcECR-B* transcripts (the two ecdysterone receptors from *T. castaneum*) revealed similar phenotypes, specifically molting defects, premature compound eye development, abnormal wing development and lethality. The observation that *TcABCG-8A* transcript levels peak in the pre-pupal (and larval stage before quiescence) suggests that gene expression is induced by ecdysterone, as it is known for the *Drosophila E23* gene, which is one of the early puff genes induced by ecdysterone. According to one suggestion, the E23 protein acts as an ecdysteroid transporter, that counteracts hormone action by pumping ecdysterone out of the cell or into intracellular compartments [14]. In both cases this would lower the effective ecdysterone concentrations in the cytosol, preventing ecdysterone from binding to cytosolic ECRs, and hence may explain why E23 negatively regulates ecdysteroid-mediated effects. Another possibility would be that E23 acts as an importer for ecdysterone residing in the plasma membrane. However, since ABC importers have only been reported in prokaryotes, this possibility has not been seriously pursued. We are currently trying to express TcABCG-8A as a functional transporter in insect cells and hope to clarify transport properties. One important question is whether TcABCG-8A acts as a homodimer or forms a heterodimer together with another TcABC half-transporter. Interestingly, our RNAi survey failed to identify a second *TcABC* gene that shared this phenotype, suggesting that TcABCG-8A forms a homodimer.

Injection of dsRNA for *TcABCG-4C* and *TcABCH-9C* revealed lethal phenotypes either at the larval-pupal or pupal-adult molt, and adult females injected with either dsRNA laid a drastically reduced number of eggs. The closest *Drosophila* homologs of *TcABCG-4C* and *TcABCH-9C* are *CG3164* and *CG9990*, respectively, which - like the *T. castaneum* genes - are expressed in all developmental stages and in intestinal/excretory tissues and carcass. A transgenic *Drosophila* RNAi line, which silences *CG3164* expression, exhibits a semi-lethal phenotype, whereas the RNAi line that silences *CG9990* is lethal [55]. While the *Drosophila* genes have not been analyzed in great detail, there are commonalities in the observations that have been made. The most striking of these is that lethality is due to a significant loss of water, as the insects showed pronounced signs of desiccation. Loss of waterproofing was most obvious for the intersegmental cuticle of pupae, where fluid bleeding was visible even by the unaided eye (see Figure 2I).

As waterproofing is largely due to the presence of lipids in the outer epicuticle, we hypothesize that both ABC proteins are directly or indirectly involved in the transport of lipids from epidermal cells to the cuticle. In line with the first interpretation, human ABCG transporters have been implicated in lipid-trafficking mechanisms [56]. However, ABCG transporters such as the ‘White’ protein have also been shown to transport important regulators of cell and tissue function such as cGMP, which could indirectly affect lipid transport [57]. Cuticular lipids are believed to be synthesized in oenocytes that are required to mobilize and process lipids from the fat body. In *T. castaneum* adults they mainly comprise neutral lipids such as n-alkanes (C_25_-C_31_) and to lower amounts 3-methylalkanes (C_26_-C_32_) and internally branched mono (C_27_-C_32_) and dimethylalkanes (C_29_-C_31_) [58], and the cuticle composition is similar in larvae [59]. To detect lipids in the cuticle and fat body, we stained with Nile red which reacts with neutral lipids such as n-alkanes, triglycerides and cholesterol esters. When comparing control and *TcABCG-4C-*dsRNA injected larvae, we observed a significant reduction in Nile red staining within the fat cells of larvae injected with *TcABCG-4C-*specific dsRNA. The loss of neutral lipids (presumably triglycerides) in the fat body indicates lipid mobilization as a consequence of starvation induced by developmental arrest. This conclusion is drawn from the finding that we obtained similar results, when we assessed Nile red staining after silencing non-ABC genes known to arrest growth and induce starvation, for example, genes encoding peritrophic matrix proteins (data not shown). In this case, we did not observe desiccation. Therefore, desiccation is likely due to the absence of specific ABC transporters involved in transport of cuticular lipids. Indeed, the lack of lipid staining in the epicuticle of larvae injected with *TcABCG-4C*-specific dsRNA suggests involvement of this ABC transporter in lipid transport. The identity of the lipid detected in the epicuticle is unknown, because Nile red stains a variety of neutral lipids. The detected signal may result from n-alkanes, but could also indicate the presence of triacylglycerides in the epicuticle, which is not easy to detect by standard GC-MS. Indeed, cuticular triacylglycerides have recently been identified in the parasitic wasp *Lariophagus distinguendus*[60]. Taken together, we provide first indication that two ABC transporters may be involved in the transport of cuticular lipids.

## Conclusions

In this study, we identified 73 ABC transporter genes in the genome of the model beetle *T. castaneum*, a common pest of stored agricultural products. In a large-scale RNAi screen, we systematically knocked-down the expression of all putative *TcABC* genes and monitored the effects on growth, development and reproduction. We found ten *TcABC* genes that are required for proper molting, cuticle differentiation and/or egg development. The results from our study provide new insights into the physiological function of ABC transporters in insects. Most notably, we identified *TcABC* genes that are potentially involved in the transport of ecdysteroids, cuticular lipids and eye-pigments. In addition, two genes that encode ABC proteins known to control protein biosynthesis may be suitable targets for RNAi-based strategies of future pest control regimes. Insecticide resistance has become a serious problem in controlling insect pests that cause crop loss and health problems. ABC transporters are known to contribute to insecticide resistance as they protect against xenobiotics and their metabolites. The coleopteran ABC transporter superfamily is significantly larger than in other insect orders, which may contribute to the evolutionary diversity of beetles and increase the genetic capacity to defend against xenobiotic stress. Thus, the improved capability to cope with xenobiotics may have contributed to the great success of the coleopteran order, which lists more species than any other group in the animal kingdom.

## Competing interest

The authors declare no conflict of interest.

## Authors’ contributions

GB and HM designed research; GB performed most of the research; TK carried out the studies on *TcABCG-9A and TcABCG-4C*; GB and HM analyzed data; and GB, ML and HM wrote the paper. All authors read and approved the final manuscript.

## Supplementary Material

Additional file 1 **Table S1.** List of primers used in this study^1^. Click here for file

Additional file 2 **Table S2.** Subfamilies of *ABC* genes in different insect species. Click here for file

Additional file 3 **Figure S1.** qPCR to evaluate the knock-down of *TcABC* transcripts by RNAi. dsRNA specific for different *TcABC* genes was injected into penultimate instar larvae (PL), pre-pupae (PP) or adults (A). Total RNA was prepared three days after the injections. Mean normalized expression values were determined by qPCR with primers specific to the indicated target gene by comparing CT values of the respective target gene and the reference gene TcRPS6. As a control, the mean normalized expression after injection of dsRNA specific for the *TcABC* genes was compared with that of insects injected with dsRNA specific for *TcVer*. Click here for file

Additional file 4 **Figure S2.** Gene loci of *TcABCA-9A* and *TcABCA-9B* and ClustalW alignment of deduced amino acid sequences. (A) The gene loci of *TcABCA-9A* (GLEAN_16282) and *TcABCA-9B* (GLEAN_16281) are shown as depicted by GBROWSE at BeetleBase. (B) ClustalW alignment of TcABCA-9A and TcABCA-9B. Highly conserved or identical amino acids are highlighted in light-gray. The sequences have been confirmed by nucleotide sequencing. Click here for file

Additional file 5 **Figure S3.***TcABC* expression in intestinal/excretory tissues and in carcass. Carcass (CAR) was obtained by removing the head, the two last abdominal segments and the complete gut with adherent Malpighian tubules from a penultimate larva. Total RNA was isolated from pools of carcass and intestinal/excretory tissues (IET) and transcribed into cDNA. Triplicates of independent cDNA preparations were used as templates in qPCRs using primers specific to the respective gene. Mean normalized expression was determined by comparing CT values of the respective target gene and the reference gene TcRPS6. Error bars indicate standard errors. Panel (A) shows *TcABC* genes with comparably low transcript levels, while panel (B) shows *TcABC* genes with higher transcript levels. Click here for file
