# Supplementary material for: Functional analysis of the ATP-binding cassette (ABC) transporter gene family of Tribolium castaneum
Source: BMC Genomics. 2013 Jan 16;14:6. doi: 10.1186/1471-2164-14-6 (PMC3560195; doi:10.1186/1471-2164-14-6)
Supplement: Additional file 1 — Table S1. List of primers used in this study1. [file 1471-2164-14-6-S1.docx]

Table S1 List of primers used in this study^1^

| Name | Forward 5’ → 3’ | Reverse 5’ → 3’ |
| --- | --- | --- |
| TcABCA-9A | GCGCATCTTTCTACGAAGAC | TACTGTTGACGGCAAGACAC |
| TcABCA-9A-T7 | TAATACGACTCACTATAGGGGAGGAATCTGATGCTTCAC | TAATACGACTCACTATAGGTTGGGAGGCTTATGGAAATG |
| TcABCA-9A-T7-2 | TAATACGACTCACTATAGGCTACCTCGTGAGGTACTATG | TAATACGACTCACTATAGGTCTCATTGGGAGGCTTATGG |
| TcABCA-9B | CATCTTTCCGCCACGACTTG | GGAAACGTCTTACTCTGATG |
| TcABCA-9B-T7 | TAATACGACTCACTATAGGTGTCGTCCGGTTCATGTTAC | TAATACGACTCACTATAGGAGTGTTGCCAGTGAGATTCC |
| TcABCA-9B-T7-2 | TAATACGACTCACTATAGGCTACCTCGTGAGGTACTATG | TAATACGACTCACTATAGGTCTCATTGGGAGGCTTATGG |
| TcABCB-5A | AACAGCACCTGCCACTGTTG | CTTCGCGATAAACACTACCC |
| TcABCB-5A-T7 | TAATACGACTCACTATAGGCGACAAACCGGTGCCCTATC | TAATACGACTCACTATAGGGGCCTGATTCATGTACACTC |
| TcABCB-5A-T7-2 | TAATACGACTCACTATAGGTCGAAGCTGAACGTTACGAC | TAATACGACTCACTATAGGCCTAAAGGGACTGAGAGTTG |
| TcABCC-4A | TGGGTGAAAGTGTCCAATCG | GAGGCAAATCCAAGGCAAAC |
| TcABCC-4A-T7 | TAATACGACTCACTATAGGATTTGCCTCGTGCTG | TAATACGACTCACTATAGGGTCTTCGTTGTCGCG |
| TcABCC-4A-T7-2 | TAATACGACTCACTATAGGCCTCAACGGACTCAC | TAATACGACTCACTATAGGTGACGCCTGATGTAG |
| TcABCC-5H | AATGCCAACGGAGGTGAAAG | CCACAGTTTGTGGCAGAATC |
| TcABCC-5H-T7 | TAATACGACTCACTATAGGGCCAACGGAGGTGAAAGAAC | TAATACGACTCACTATAGGGTACCACTTGCACCCAACTG |
| TcABCC-9A | TGGAGGAGGCGAAGAAGGTC | ATGCGTCCAACCGGAGTTAC |
| TcABCC-9A-T7 | TAATACGACTCACTATAGGAAGTCGCAACTTAGCGGAAC | TAATACGACTCACTATAGGTTCGCCCTTGACCTTCTTCG |
| TcABCC-9A-T7-2 | TAATACGACTCACTATAGGTTGGCGCCTCAGTTAGGTTG | TAATACGACTCACTATAGGACCTCAAACAGGCAATAAAC |
| TcABCE-3A | GGTCTGAGCTGCAGAATTAC | AAATTGCCGGCCAACATTCG |
| TcABCE-3A-T7 | TAATACGACTCACTATAGGACTATGCCATTCTCCGTTCG | TAATACGACTCACTATAGGTCGAAGGGTACTCGTAATGG |
| TcABCE-3A-T7-2 | TAATACGACTCACTATAGGTTGGAAGACGACCTGAAAGC | TAATACGACTCACTATAGGCAGGCAAATCTCTGCAACTC |
| TcABCF-2A | AGGACGAAGGGCTTGTTACG | TTTGCGACTGGGAGACTGTG |
| TcABCF-2A-T7 | TAATACGACTCACTATAGGAACTCACCCACAAGGAGAAG | TAATACGACTCACTATAGGTTTGCGACTGGGAGACTGTG |
| TcABCF-2A-T7-2 | TAATACGACTCACTATAGGGAAGGGCTTGTTACGAAAGG | TAATACGACTCACTATAGGTACCGGACTCATGTTCCTTC |
| TcABCG-4C | AATCCGAGCGTGAAGACATC | CGCAGGCAAATTCTCTTTGG |
| TcABCG-4C-T7 | TAATACGACTCACTATAGGGAAGCTGGTCGAGGCGATTG | TAATACGACTCACTATAGGCGCAGGCAAATTCTCTTTGG |
| TcABCG-4C-T7-2 | TAATACGACTCACTATAGGTTCTTGGCACCGGTCATGTC | TAATACGACTCACTATAGGTGGTGGTGGGCGATTTAAAG |
| TcABCG-8A | TCTTCGGTGAGTTCGGAGAC | TGTTCTGGTAGGCGTAGTGC |
| TcABCG-8A-T7 | TAATACGACTCACTATAGGTCGCAAGAGCAAGGACTACC | TAATACGACTCACTATAGGGGCCACGAGAAGTAGCAATC |
| TcABCG-8A-T7-2 | TAATACGACTCACTATAGGTCTGGCTGACCTGGATGAAG | TAATACGACTCACTATAGGCGAGCAAGAAGGCTAGTAAG |
| TcABCG-9A | GGATTGGGATCAATGGAGAG | ATAGCTGCGGGCACGGAATC |
| TcABCG-9A-T7 | TAATACGACTCACTATAGGAAACGCTAGCAACGACGCCC | TAATACGACTCACTATAGGTGGTCCTCAGTTTGTAAATC |
| TcABCG-9A-T7-2 | TAATACGACTCACTATAGGATTCCGTGCCCGCAGCTATG | TAATACGACTCACTATAGGCTTGAGTGTCTGCAGTTAGG |
| TcABCG-9B | TCGAGGCTGCCACTACTGAG | AACGCTACGGGAGACACAGG |
| TcABCG-9B-T7 | TAATACGACTCACTATAGGTGCCGATTATTTCATCCAAC | TAATACGACTCACTATAGGCCAACACGAGGCTTTATACG |
| TcABCG-9B-T7-2 | TAATACGACTCACTATAGGTGTGTCTCCCGTAGCGTTTC | TAATACGACTCACTATAGGCTTTAGGGCACGGCAGATCG |
| TcABCH-9C | TGGAGTCCTCGGAAATCAAC | CGAACGAGCACTCCTGGTAG |
| TcABCH-9C-T7 | TAATACGACTCACTATAGGAGTCTAGCGACGCTCAACTG | TAATACGACTCACTATAGGAGGAGGTCATGTCGGAGCAG |
| TcABCH-9C-T7-2 | TAATACGACTCACTATAGGGTGAAGACGACAAGCTGAAG | TAATACGACTCACTATAGGGTCGCTGAGGACGTGGTTGG |
| TcVER-T7 | TAATACGACTCACTATAGGTTGGTGGACCAAGTAATGAT | TAATACGACTCACTATAGGGCCATTTCGTGATCAGCGAG |
| TcRPS6 | AGATATATGGAAGCATCATGAAGC | CGTCGTCTTCTTTGCTCAAATTG |
| TcRosy | CGATCCGGTGCATGCTAGAC | CCCGTCTCCCAATTGTAGCC |
| TcRosy-T7 | TAATACGACTCACTATAGGGCGCCGACATTAAACTCTAC | TAATACGACTCACTATAGGGCGTGAAATCGCCTTCTTTG |
| TcSepia | GGCAAACTCCGGTTATACAG | GTACTCCAGCCCTGGATGAC |
| TcSepia-T7 | TAATACGACTCACTATAGGGCCAACGTGTTATCCTAGTC | TAATACGACTCACTATAGGCATGTAACCGGTGGGCGTTG |

**^1^**This list does only include primers for genes whose knock-down caused developmental phenotypes.
