# Supplementary material for: Functional analysis of the ATP-binding cassette (ABC) transporter gene family of Tribolium castaneum
Source: BMC Genomics. 2013 Jan 16;14:6. doi: 10.1186/1471-2164-14-6 (PMC3560195; doi:10.1186/1471-2164-14-6)
Supplement: Additional file 2 — Table S2. Subfamilies of ABC genes in different insect species. [file 1471-2164-14-6-S2.docx]

**Table S2** Subfamilies of *ABC* genes in different insect species

| **Species**  **ABC subfamily** | *Drosohila melanogaster* | *Anopheles gambiae* | *Apis mellifera* | *Bombyx*  *mori* | *Tribolium castaneum* |
| --- | --- | --- | --- | --- | --- |
| A | 10/10 | 7/9 | 3 | 9/6 | 10/9 |
| B | 10/8 | 5/5 | 7 | 9/8 | 6/6 |
| C | 12/14 | 13/13 | 9 | 15/15 | 35/31 |
| D | 2/2 | 2/2 | 2 | 2/2 | 2/2 |
| E | 1/1 | 1/1 | 1 | 1/1 | 1/1 |
| F | 3/3 | 3/3 | 3 | 3/3 | 3/3 |
| G | 15/15 | 11/16 | 15 | 12/13 | 13/13 |
| H | 3/3 | 3/3 | 3 | 2/3 | 3/3 |
|  |  |  |  |  |  |
| Total | 56/56 | 45/52 | 43 | 53/51 | 73/68 |
| Reference | [[5](#_ENREF_5)]/ [[20](#_ENREF_20)] | [[27](#_ENREF_27)]/[[20](#_ENREF_20)] | [[20](#_ENREF_20)] | [[21](#_ENREF_21)]/[[20](#_ENREF_20)] | This study/[[20](#_ENREF_20)] |
