# Supplementary figures and images for: Functional analysis of the ATP-binding cassette (ABC) transporter gene family of Tribolium castaneum
Source: BMC Genomics. 2013 Jan 16;14:6. doi: 10.1186/1471-2164-14-6 (PMC3560195; doi:10.1186/1471-2164-14-6)

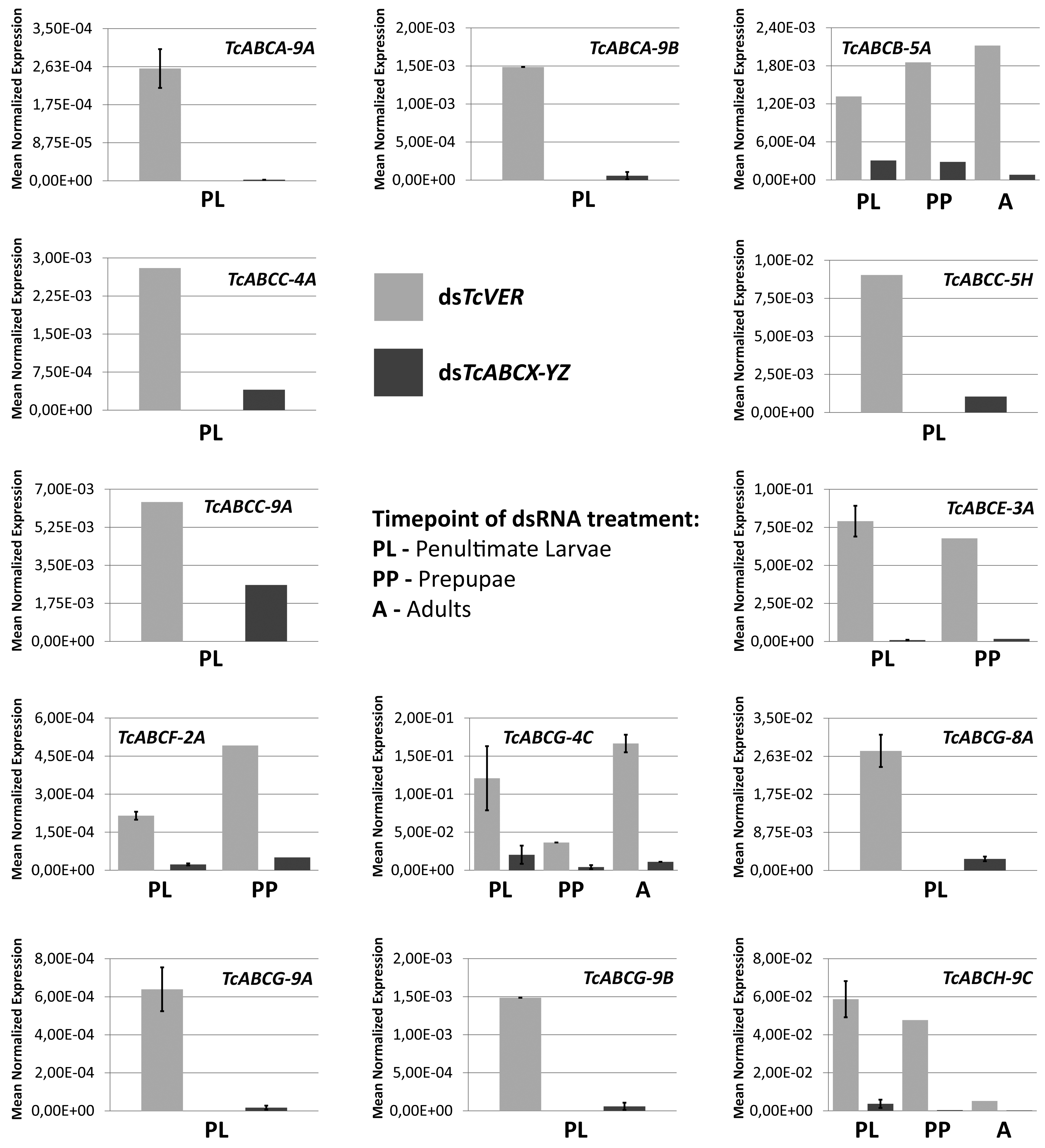

Supplement: Additional file 3 — Figure S1. qPCR to evaluate the knock-down of TcABC transcripts by RNAi. dsRNA specific for different TcABC genes was injected into penultimate instar larvae (PL), pre-pupae (PP) or adults (A). Total RNA was prepared three days after the injections. Mean normalized expression values were determined by qPCR with primers specific to the indicated target gene by comparing CT values of the respective target gene and the reference gene TcRPS6. As a control, the mean normalized expression after injection of dsRNA specific for the TcABC genes was compared with that of insects injected with dsRNA specific for TcVer. [file 1471-2164-14-6-S3.tiff]

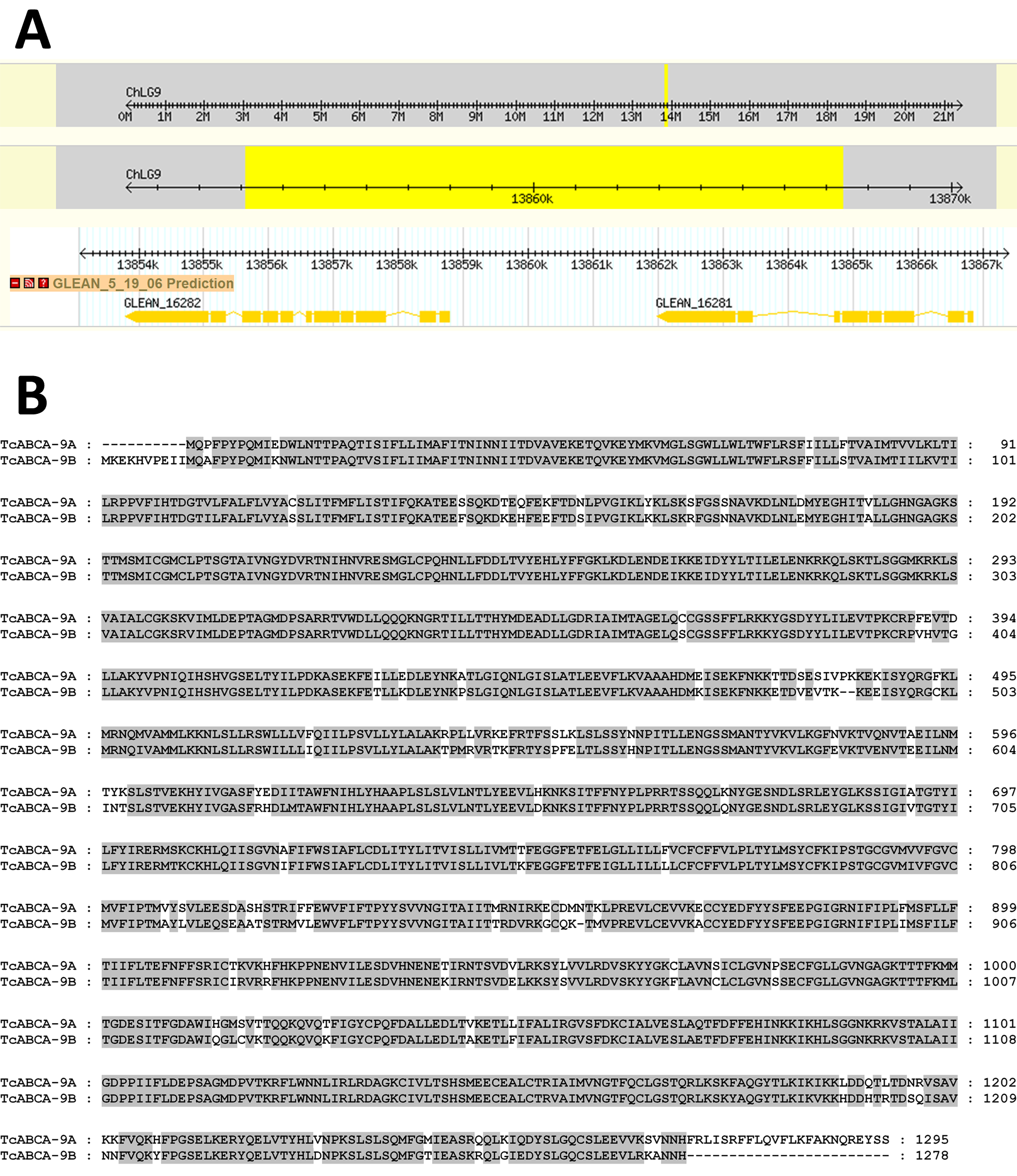

Supplement: Additional file 4 — Figure S2. Gene loci of TcABCA-9A and TcABCA-9B and ClustalW alignment of deduced amino acid sequences. (A) The gene loci of TcABCA-9A (GLEAN_16282) and TcABCA-9B (GLEAN_16281) are shown as depicted by GBROWSE at BeetleBase. (B) ClustalW alignment of TcABCA-9A and TcABCA-9B. Highly conserved or identical amino acids are highlighted in light-gray. The sequences have been confirmed by nucleotide sequencing. [file 1471-2164-14-6-S4.tiff]

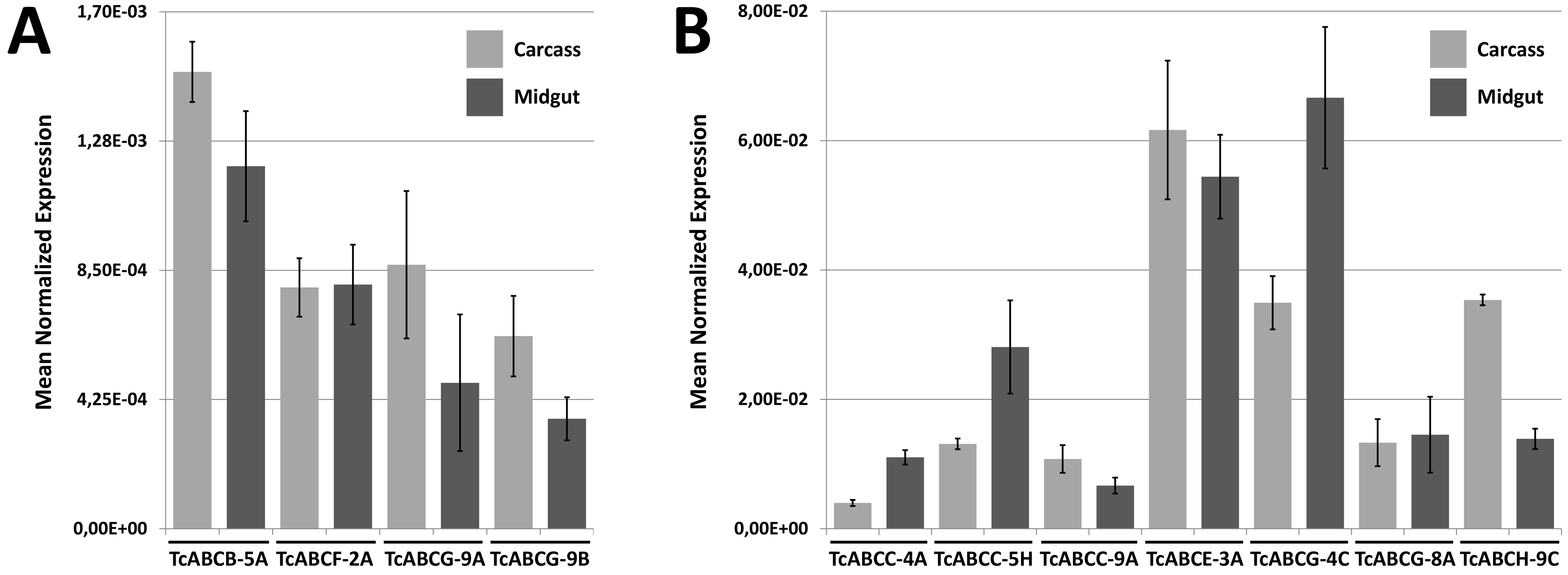

Supplement: Additional file 5 — Figure S3.TcABC expression in intestinal/excretory tissues and in carcass. Carcass (CAR) was obtained by removing the head, the two last abdominal segments and the complete gut with adherent Malpighian tubules from a penultimate larva. Total RNA was isolated from pools of carcass and intestinal/excretory tissues (IET) and transcribed into cDNA. Triplicates of independent cDNA preparations were used as templates in qPCRs using primers specific to the respective gene. Mean normalized expression was determined by comparing CT values of the respective target gene and the reference gene TcRPS6. Error bars indicate standard errors. Panel (A) shows TcABC genes with comparably low transcript levels, while panel (B) shows TcABC genes with higher transcript levels. [file 1471-2164-14-6-S5.tiff]
